# Avascular necrosis as an uncommon manifestation in glycogen storage disease type III: diagnostic and therapeutic challenges

**DOI:** 10.1093/omcr/omaf145

**Published:** 2025-08-20

**Authors:** Faiq I Gorial, Nabaa Ihsan Awadh, Sara S Khunda, Sajjad Ghanim Al-Badri, Ali Falah Alibrahimi

**Affiliations:** Rheumatology Unit, Department of Internal Medicine, College of Medicine, University of Baghdad, Iraq; Rheumatology Unit, Department of Internal Medicine, College of Medicine, University of Baghdad, Iraq; Rheumatology Unit, Department of Internal Medicine, College of Medicine, University of Baghdad, Iraq; College of Medicine, University of Warith Al-Anbiyaa, Karbala, Iraq; University of Baghdad, Alkindy College of Medicine, Baghdad, Iraq

**Keywords:** glycogen storage disease type III, avascular necrosis, multidisciplinary management

## Abstract

Glycogen storage disease type III (GSD III), or Cori disease, is a rare autosomal recessive disorder caused by a debranching enzyme deficiency, leading to abnormal glycogen accumulation. Clinical features include hepatomegaly, hypoglycemia, myopathy, and cardiomyopathy. Avascular necrosis (AVN), the death of bone tissue due to poor blood supply, is an uncommon but severe complication of GSD III. This report discusses a 19-year-old female with a known diagnosis of GSD III who developed AVN, presenting with chronic right hip pain, muscle weakness, and hypoglycemic seizures. Diagnostic challenges were resolved using imaging studies. Management included dietary adjustments to stabilize blood glucose, symptomatic treatment, lipid-lowering agents, and osteoporosis therapy to address skeletal complications. This case highlights the importance of multidisciplinary care, regular monitoring, and individualized management to address rare complications and improve outcomes in GSD III.

## Introduction

Glycogen storage disease type III, commonly known as Cori disease or Forbes disease, is an autosomal recessive metabolic disorder associated with a deficiency of the enzyme debranching enzyme (amyloid-1,6-glucosidase) [1, 2]. This results in the accumulation of structurally altered glycogen in the liver, muscles, and other tissues, leading to various clinical presentations, including hepatomegaly, hypoglycemia, myopathy, and cardiomyopathy. Avascular necrosis (AVN) or osteonecrosis is the death of bone tissue due to a lack of blood supply, which leads to bone death and possible collapse. Although AVN is commonly associated with factors such as trauma, steroid use, and alcoholism, this event in metabolic disorders such as GSD III is relatively uncommon and not well-documented [3–6].

This case report details a patient diagnosed with Glycogen Storage Disease Type III (GSD III), who later developed avascular necrosis (AVN), an uncommon association reported in patients with GSD III. This report explores the diagnostic challenges in identifying AVN in the context of GSD III and discusses the intricacies involved in managing such a complex case, where both conditions influence the clinical approach and patient outcomes.

## Case presentation

A 19-year-old female with a known diagnosis of Glycogen Storage Disease Type III (GSD III) presented to the rheumatology unit of Baghdad Teaching Hospital with a primary complaint of right hip pain persisting for three years. Her GSD III had been previously diagnosed in childhood and managed through a corn starch diet and lactose-free dairy products under the supervision of a pediatrician. She presented to our unit due to the progressive nature of her hip pain, which was exacerbated by walking and movement but relieved by rest and analgesics.

**Figure 1:**
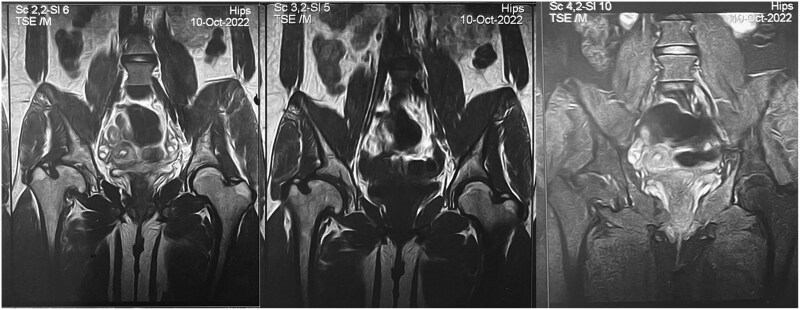
MRI images show right femoral head deformity with signs of avascular necrosis, including coxa magna, coxa vara, and a short neck. The femoral head is partially contained with acetabular remodeling. No labral tears, cysts, hip effusion, or synovial thickening. Both SIJ and lower lumbar vertebrae are normal.

She had also been experiencing symptoms of muscle weakness manifested by difficulty rising from a sitting position, and walking upstairs was particularly challenging for her. On further questioning, she reported weakness in her proximal arms when washing and combing her hair. She denied any other joint swelling, pain, or deformity, and no muscle pain or stiffness was reported. She had a history of abnormal body movements with loss of consciousness, which she described as tonic–clonic with up-rolling of the eyes and tongue biting. Low blood glucose levels preceded them, and the patients attributed them to her poor adherence to dietary starch. There was no history of other neurological problems, such as sensitivity to light, visual changes, or numbness. Other significant symptoms on review were difficulty swallowing solids and colicky abdominal pain with nausea and heartburn. She was investigated for gastrointestinal complaints and treated with proton pump inhibitors for gastritis. She didn’t have a history of steroid use and alcohol consumption, There was a history of recurrent oral ulcers. No jaundice or change in bowel habits, urine, or stool color changes were reported. She had no history of bluish discoloration of the fingers and no ocular or oral dryness.

Her family history was significant, as her two siblings were diagnosed with a similar condition in childhood and had undergone liver transplantation for advanced cirrhotic liver disease. The family had requested a genetic study, which revealed the maternal grandmother to be a carrier of the responsible gene.

On physical examination, she was found to have a painful limitation of the right hip joint motion in abduction and external and internal rotations.

Furthermore, the Brandenburg test was indicative of hip muscle weakness. A neurological exam revealed mild weakness of the left thigh muscle, power grade 4 on hip flexion, and reduced muscle power (grade 4) of the left upper arm on shoulder abduction. Her muscle tone is normal and no muscle wasting or fasciculation was observed. Reflexes were intact throughout, and the Babinski sign was downward. The abdominal exam was significant for stria and hepatomegaly; the liver span was 14 cm. No tenderness or guarding was noted.

Accordingly, she was sent for baseline investigations, including complete blood counts, inflammatory markers, liver function and renal function tests, muscle enzymes, a virology screen, and a lipid profile. A notable rise in liver enzymes was recorded with alanine aminotransferase (ALT) at 130 U/l, aspartate aminotransferase (AST) at 171 U/l, and alkaline phosphatase (ALP) at 132 U/l. Creatinine phosphokinase (CPK) levels were also elevated, measuring 1456 U/l. Her lipid profile was also abnormal, with total cholesterol at 303 and triglycerides at 307. Thyroid function was significant for subclinical hypothyroidism. The virology screen was negative, while the other tests showed no abnormalities, further assisting in excluding other potential causes for the patient’s presentation.

Imaging studies were done. An abdominal ultrasound showed a 17.6-cm-wide, hyperechoic liver that did not have any focal or space-occupying lesions. This suggests that the liver has cirrhosis. Median liver stiffness on elastography was 11, ranked within the F2-F3 range. The liver biopsy result was reviewed, which showed distorted liver architecture with fibrous bands and regenerative nodules of hepatocytes that showed prominent ballooning and swelling compressing sinusoids. Picture compatible with glycogen storage disease, cirrhotic stage. An MRI was ordered to look into the right hip pain. The results showed that the right femoral head was deformed, with a large, broad articular cortex head (coxa magna), coxa vara, and a short neck with an irregular articular cortex showing fragmentation and sclerosis. Subchondral bone marrow edema is suggestive of avascular necrosis (Fig. 1).

Accordingly, a management plan was implemented symptomatic therapy and lipid-lowering drugs. She was prescribed Alendronate tablets 70 mg once weekly, calcium supplements, atorvastatin 20 mg daily, and low-dose aspirin 75 mg.

## Discussion

Glycogen storage disease type III (also called glycogen debranching enzyme deficiency, GDE, or Cori-Forbes disease) is a rare inherited disorder and is classified into four types depending on the age of onset, with type Ib being the most common. This leads to hepatomegaly, hypoglycemia, and myopathic deterioration, which mainly starts in childhood [7]. The co-existence of avascular necrosis (AVN) with GSD III, as observed in this case report of a 19-year-old female patient, highlights an unusual association with the disease. Avascular necrosis (AVN) is a pathogenetic process characterized by vascular restriction resulting in bone ischemia, infarction, and ultimately structural failure. AVN is commonly associated with factors such as trauma, steroid use, and alcoholism. The association between Glycogen Storage Disease Type III (GSD III) and avascular necrosis (AVN) remains unclear and has not been previously well-documented. While AVN is more commonly associated with GSD I due to bone marrow edema caused by myeloid dysfunction and neutropenia, such a mechanism is absent in GSD III as it does not involve myeloid dysfunction [8]. The absence of a clear theoretical basis to directly link GSD III with AVN suggests that the association in this case may be coincidental. However, the patient’s progressive right hip pain, proximal muscle weakness, and elevated creatine phosphokinase (CPK) levels underscore the importance of clinical vigilance regarding musculoskeletal complications in GSD III patients. This emphasizes the need for further investigations to rule out other common causes of AVN.

The abdominal ultrasound suggests that the liver has cirrhosis; Viral liver cirrhosis has been identified as a risk factor for AVN. However, serological tests in this case did not show evidence of viral liver cirrhosis, this possibility cannot be entirely ruled out and warrants further investigation [9].

Another consideration is the potential exposure to alternate therapies, such as steroids or heavy metals, which could predispose to AVN. Although the patient denied a history of steroid use, the presence of striae raises the possibility of undiagnosed or undocumented steroid exposure.

Identifying AVN in GSD III patients highlights the need for further study. Future research should focus on the molecular mechanisms linking GSD III to musculoskeletal complications and examine the impacts of lifestyle and medication history. Longitudinal studies observing the progression of GSD III and interdisciplinary collaboration are essential for developing a comprehensive understanding and management of the disease.

Imaging studies for diagnosing AVN are fundamental. MRI of the right hip showed classical features (coxa magna, coxa vara, irregular articular cortex with fragmentation and sclerosis) indicative of AVN and subchondral bone marrow edema. These results confirm the diagnosis and assess the grade of AVN to decide the appropriate treatment [10].

The management of GSD III consists mainly of maintaining normoglycemia with dietary interventions, such as a cornstarch diet and lactose-free dairy products to prevent hypoglycemia. For symptomatic therapy and treatment of underlying metabolic abnormalities in AVN, Alendronate (a bisphosphonate that inhibits bone resorption and is used for treating osteoporosis) was added; it may help in AVN by decreasing turnover and preserving bone mass. Calcium supplements were prescribed to support general skeletal health, and atorvastatin was started to treat dyslipidemia, reducing cardiovascular risk. Low-dose aspirin was included for its anti-platelet activity, preventing thrombotic events that may worsen AVN [11, 12].

This case highlights the role of all specialties involved in managing GSD III, including pediatricians, rheumatologists, orthopedists, and metabolic specialists. Continued follow-up is crucial to monitor liver function, muscle strength, and metabolic control while individualizing pharmacotherapy. The family history of GSD III is also striking, as two older siblings had to undergo liver transplantation for end-stage cirrhosis, illustrating the genetic and progressive nature of the disease and necessitating early intervention and genetic counseling [13, 14].

## Conclusion

This case describes the challenging diagnosis and therapeutic management of AVN in a patient with GSD III, indicating a high index of suspicion for such complications. An ongoing surveillance approach to screen for skeletal changes is critical. Additional investigation into the pathophysiology connecting GSD III with AVN might help develop focused therapies and preventive measures to enhance affected individuals.
